# Three-Dimensional Printing Technology in Orthodontics for Dental Models: A Systematic Review

**DOI:** 10.3390/children9081106

**Published:** 2022-07-23

**Authors:** Ioannis A. Tsolakis, Sotiria Gizani, Nearchos Panayi, Georgios Antonopoulos, Apostolos I. Tsolakis

**Affiliations:** 1 Department of Orthodontics, School of Dentistry, Aristotle University of Thessaloniki, 54124 Thessaloniki, Greece; 2 Department of Paediatric Dentistry, Dental School, National and Kapodistrian University of Athens, 15772 Athens, Greece; sotiriagizani@gmail.com; 3 Department of Orthodontics, School of Dentistry, European University of Cyprus, Nicosia 1516, Cyprus; dr.panayi@cytanet.com.cy; 4 School of Medicine, National and Kapodistrian University of Athens, 11527 Athens, Greece; 5 Private Dental Lab “FN Orthodontics”, 11527 Athens, Greece; antonopoulosgr@gmail.com; 6 Department of Orthodontics, School of Dentistry, National and Kapodistrian University of Athens, 15772 Athens, Greece; apostso@otenet.gr; 7 Department of Orthodontics, Case Western Reserve University, Cleveland, OH 44106, USA

**Keywords:** 3D printing, three-dimensional printing, accuracy, dental models, dental casts, systematic review, orthodontics, dentistry

## Abstract

Background: Three-dimensional printing technology is an additive manufacturing technology that is used to reconstruct 3D objects. In the last decade, it has been rapidly involved in dentistry and in orthodontics. This article aims to review the literature and present the accuracy of different 3D printer types and any factors that could affect the 3D printing of dental models in the orthodontic field. Methods: The search strategy of this systematic review included keywords in combination with MeSH terms in Medline, Scopus, and Cochrane Library until June 2022 and only in English. Results: Eleven articles were selected for our study. All the articles were in vitro prospective studies, and they presented a low risk of bias. The results suggested that the accuracy of a printed dental cast can be affected by the different types of 3D technologies, the dental cast’s base design, and the printing materials. The accuracy appears to not be affected by the layer height and the position of the model on the building template. Conclusions: According to this systematic review, all different types of 3D technology can produce clinically accepted results for orthodontic purposes. There is a need for more studies to clarify the accuracy and added value of 3D printing technology in orthodontics.

## 1. Introduction

Advanced technology has been rapidly involved in dentistry and, more specifically, orthodontics. Three-dimensional printing is one of the cutting-edge technologies in the manufacturing industry. One of the very first uses of three-dimensional printers in orthodontics was to create dental casts. The intraoral scanner gave the ability to dentists to take a dental impression without the discomfort feeling that traditional impressions were causing to patients. The use of intraoral scanners resulted in a three-dimensional image that could be printed [1,2,3,4].

The very first three-dimensional (3D) printer was introduced by Charles Hull in 1986. The same year Hull found stereolithography (SLA) and developed the first 3D printing system [5,6,7,8]. Four years later, fused deposition modeling (FDM) was introduced by Scott Crump [9]. SLA printing technology became more popular in the dental field because of its accuracy and rigidity. Nowadays, the most commonly used three-dimensional printers are the Laser-SLA, the Direct Light Processing (DLP), and the Liquid Crystal Display (LCD). Those three types of printers consist of a VAT and building platform. On the VAT liquid photopolymer resin is placed in order to create the printing model. Another type of printer that is becoming popular is the Fused Filament Fabrication (FFF). These printers mainly consist of an extruder and a building plate. Through the extruder, a plastic-based material is heated in order to build up the model on the plate. Lastly, a very popular 3D printing technology is the PolyJet photopolymer (PPP). The PolyJet printers consist of a material container, inkjet print heads, and a build platform [10,11,12,13,14].

Accuracy consists of precision and trueness. Precision describes how close repeated measurements are to each other. Therefore, a printer with higher precision correlates to a more repeatable and consistent print. Trueness describes how far the measurement deviates from the actual dimensions of the measured object. Therefore, a printer with high trueness indicates that the printer delivers a result that is close to or equal to the actual dimensions of the digital 3D object [15,16,17,18,19,20].

The introduction of three-dimensional printing technology to the dental field gave the ability to the practitioners to deliver low-cost appliances directly to the patients bypassing the dental lab. This article aims to systematically review the literature and present the accuracy of different 3D printer types and other factors that could affect the 3D printing of dental models in the orthodontic field.

## 2. Materials and Methods

**Protocol and registration**

The protocol for this present systematic review was registered on the Open Science Forum Database following the Prisma-P guidelines 1 (Protocol: 10.17605/OSF.IO/QGPD7, accessed on 9 July 2022).

**Eligibility criteria**

The following selection criteria were applied for the review.

1. Study design: randomized or non-randomized, prospective or retrospective in vitro studies.
2. Participants: dental models.
3. Interventions: studies that printed dental models for orthodontic purposes.
4. Comparisons: comparisons were made between the original stl file and the printed outcome.
5. Outcomes measures: any difference between the original file and the printed outcome.

**Information sources, search strategy, and study selection**

This critical review was conducted by using the following keywords in the search strategy “3D printing”, “orthodontics”, and “dental model”. Those keywords were combined with the following Medical Subject Heading (MeSH terms): “Printing, Three-Dimensional” [Majr], “Models, Dental” [Majr], “Dental Cast” [Majr], “Orthodontics” [Majr]. The databases used for the electronic search were Cochrane Library, Medline (PubMed), and Scopus. Additionally, a hand search was performed. There was a selection of only English-written language articles without any limit to the publication period. Studies that included personal opinions were excluded. The search was conducted for studies published by June 2022. The search strategy for PubMed is presented in Table 1.

Studies were selected in duplicate and independently by two authors (I.A.T. and N.P.). Any possible inconsistency was resolved through discussion with the other two authors (S.G. and A.I.T.). They were not blinded while identifying the authors of the studies, their institutions, or their research findings. After identifying potentially relevant studies by title, the authors read the abstract and ruled out ineligible studies. A manual search of eligible study references was later performed to find additional articles that could not be found by searching the database. Finally, after reading the articles in full, the choice was made according to our inclusion and exclusion criteria (Table 2).

**Data items and collection extraction and management**

Two review authors (I.A.T. and N.P.) extracted the data independently and in duplicate. The data that were extracted included participants, intervention, outcomes, methods of outcome assessment, results, and conclusion. When the authors did not have access to the missing data, they reported and analyzed only the existing data.

**Risk of bias/Quality assessment in individual studies**

A quality assessment of the methodology of the included studies was performed using the quality assessment of the ACROBAT-NRSI tool of Cochrane to perform the assessment of the studies’ risk of bias and applicability concerns. Each domain was assessed and ranked as high risk, low risk, or unclear based on the following:

1. Low risk of bias if all key domains of the study were at low risk of bias.
2. Unclear risk of bias if one or more key domains of the study were unclear.
3. High risk of bias if one or more key domains were at high risk of bias.

## 3. Results

The initial data search resulted in 3703 studies. Out of all these papers, only 52 were selected by the title of the study. Afterward, each selected article was fully evaluated by two different reviewers by reading the entire script. Finally, 11 papers were selected for the present critical review.

**Figure 1:**
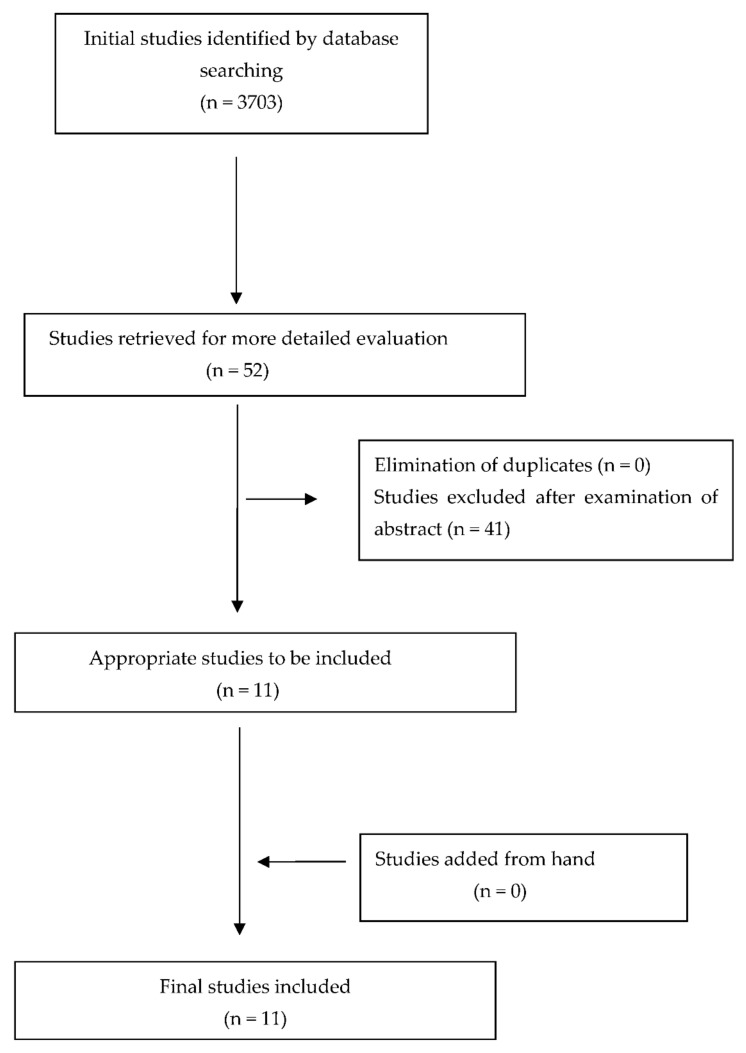
Flow diagram—selection of studies.

All the final selected articles were in vitro prospective studies. Eight of these articles evaluated the accuracy of 3D printing technology in creating dental models. Three out of all articles studied other parameters as well that might affect the printing accuracy such as the resin type, the position of the models, and their design. Two articles focused on the accuracy effect of layer height in 3D printed models, and one article evaluated the effect of body design of the printed cast on the printed accuracy [21,22,23,24,25,26,27,28,29,30,31]. The procedure of article selection is presented on a flow diagram (Figure 1), and data are briefly presented in Table 3.

**Risk of bias within studies**

The seven criteria for the non-RCT studies were: bias due to confounding, bias in the selection of participants into the study, bias in the measurement of interventions, bias due to departures from intended interventions, bias due to missing data, bias in measurement outcomes, and bias in the selection of the reported result. All studies presented a low risk of bias in all measurements (Table 4).

## 4. Three-Dimensional Printing Technologies

**Figure 2:**
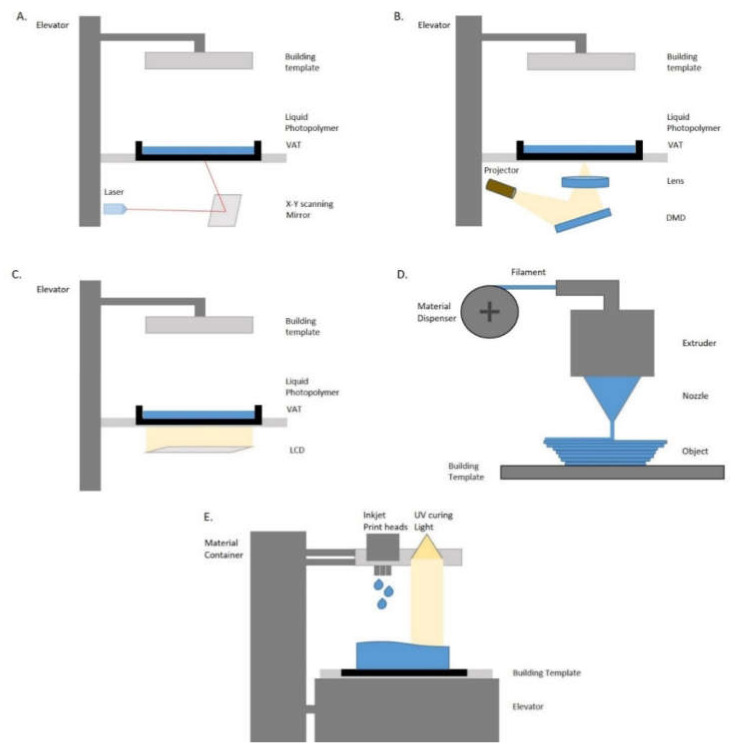
Three-dimensional printing technologies. (**A**) Laser-SLA, (**B**) DLP, (**C**) LCD, (**D**) FFF, and (**E**) PPP.

Different 3D printing technologies were assessed in the included studies. According to our search, the types of printing technology that have been tested in the literature were Laser-stereolithography (laser-SLA), Direct Light Processing (DLP), Liquid Crystal Display (LCD), Fused Filament Fabrication (FFF), and PolyJet Photopolymer technology (PPP) (Figure 2).

Laser-SLA 3D printing technology is characterized by the UV laser that is used to build the printing model. The process starts by positioning the building platform in the tank of a liquid photopolymer by keeping a distance of a layer height. The UV laser fabricates the upcoming layer by selectively curing the photopolymer resin. Once the resin is cured it becomes solid. The laser beam is moving in a predetermined path by using a set of mirrors. Those mirrors are called galvos. At the end of the printing process, the printing object is not fully cured. This is the reason that the object requires further UV light exposure. Before the post-processing UV light cure, the object needs an ultrasonic bath with isopropyl alcohol in order to get rid of the resin remnants.

DLP, like Laser-SLA, is a type of vat polymerization. Vat polymerization technologies use a liquid photopolymer resin which is cured by a light source. DLP technology is a 3D additive manufacturing technology that is based on a digital light projector. The DLP projector has the ability to flash an image of a layer at once. Therefore, all points of a layer can be cured at the same it. This characteristic makes the DLP printing technology much faster than Laser-SLA. The light source of the DLP printer is a LED screen which is composed of a digital micromirror device (DMD). This device contains millions of small micromirrors that direct the light and form the pattern of a layer onto the bottom of the resin tank. At the end of the printing process, like Laser-SLA, the model needs to be washed and post-cured.

LCD is a 3D printing technology that is based on the same scientific information as Laser-SLA and DLP printers (vat polymerization technology). The LCD printers use liquid crystal display as a light source. In LCD 3D printers, the light shines in parallel, coming through the LCD panels onto the build area. Moreover, the light is not expanded using any lens or other device. Hence, pixel distortion is not an issue when working with an LCD 3D printer. The main difference from the Laser-SLA printers is that LCD printers are faster. LCD printers are as fast as DLP printers, but LCD printers are more affordable because of the low-cost manufacturing materials that it is needed to build them up. This is the main reason those printers are becoming more popular.

FFF is an additive manufacturing process. During this process, a thermoplastic material is forced through an extruder (heated nozzle) to create an object. Once the first layer is added, the building platform is moved into a layer distance in order to create the second layer. This process continues by adding layer by layer till the final fabrication of the printed object. The most common thermoplastic materials that have been used in FFF printing technology are polylactic acid (PLA) and acrylonitrile butadiene styrene (ABS). The first one is known for its excellence in detailing and the second one for its durability.

PPP is a 3D printing technology that works like an inkjet regular printer. It builds parts by jetting thousands of photopolymer droplets onto a build platform and solidifying them with UV light. For this type of technology, photopolymer resin is used. Before printing starts, the resin is poured into the container in order to be heated. Once resin reaches the right viscosity, printing starts with the carriage moving across the *x*-axis, across the build platform. During this procedure, the print heads selectively jet the resin into the build platform. Once the resin is jetted, UV light cures it. After a single layer is complete, the build platform moves a layer down in height, and the process continues until the object is printed.

According to our search, there are eight articles in the present literature that tried to find which of these 3D printed technologies can deliver an accurate result in general. In 2014, Hazeveld et al. compared the accuracy of DLP and PPP printers. They found that the PPP printer is more accurate than the DLP printer [30]. Four years later, Dietrich C.A. et al. evaluated the differences in trueness and precision of PolyJet and SLA printers [29]. They found that PPP could deliver better trueness, but Laser-SLA printers were better precision-wise. In the same year, Kim et al. compared the Laser-SLA, the DLP, the FFF, and the PPP printers [21]. Their research suggested the precision of the PolyJet printer was the best, followed by the DLP printer, the SLA printer, and the last one was the FFF printer. The PPP was the best due to trueness as well, followed by the SLA printer, DLP printer, and last, the FFF printer. In 2018, there were two different studies that evaluated the accuracy of PPP and DLP printers compared to dental stone models. The first one was conducted by Brown et al., and their result suggested that there was a statistically significant difference between the two printers and the dental casts, but there was not a clinically significant difference [26]. The second study was performed by Park et al., and their result suggested that the stone model is more reliable than the two printers model [27]. In 2020, Pereira et al. looked over the difference in the accuracy between the DLP, FFF, and PPP printers [22]. They concluded that PPP showed the best accuracy, followed by DLP and then FFF. Lastly, in the same year, Akyalcin S. et al. compared DLP, SLA, and PPP printing technology [23]. Their results suggested that DLP and PPP printers had the same accuracy, while SLA was less accurate in printing dental models. In 2022, Giudice et al. evaluated the accuracy of entry-level LCD printers compared to Laser-SLA printers. They found that LCD printers are not as accurate as Laser-SLA printers, but their difference is not clinically significant [31].

## 5. Printing Layer Thickness

**Figure 3:**
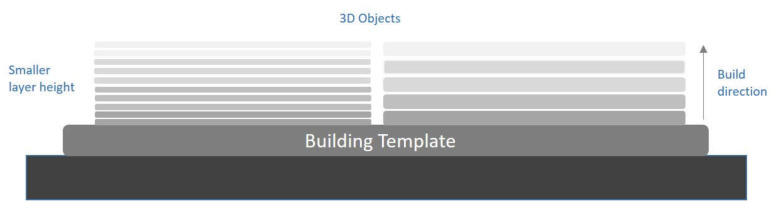
Layer height in 3D building technology.

Three-dimensional printing is an additive manufacturing technique; this means that the object will be printed layer by layer. These layers have a thickness. Layer thickness refers to measuring the layer height of each subsequent addition of the material in additive manufacturing or the method used in 3D printing where layers are stacked. It is one of the most important technical characteristics of all 3D printers. The layer height corresponds to the vertical resolution of the *z*-axis. Printing speed and printing time are generally affected by the number of layers required to create an object. A 3D printed object of a given height takes longer to create the thinner the layer is. Typically, the minimum and maximum layer heights for 3D printers are 16 mm and 150 mm, respectively. Before starting the 3D printing of a 3D file, all 3D printers have the option to adjust this setting. The printing time required and the results of a smoother surface are also greatly determined by layer height (Figure 3).

In the present literature, there were two studies that looked over the effect of layer thickness on the printing accuracy of a dental model. In 2018, Sherman S.L. et al. compared the layer height of 50 microns to the layer height of 100 microns by using a DLP printer [24]. All measurements were based on linear measurement in mesiodistal, incisal-gingival, intercanine, intermolar, and arch depth terms. They concluded that there was no difference between those different layer height models. A year later, Loflin W.A. et al. compared three different layer heights (25, 50, and 100 microns) to stone models [25]. For this study, a Laser-SLA printer was used. They found that there was no difference between the different heights, and there was a high correlation between the printed models to the stone models.

## 6. Position on Building Template

All the different types of three-dimensional printed technologies are composed of a building template. This template is the base where the object is printed layer by layer. In Laser-SLA, DLP, and PolyJet, this building platform is metallic, while in the FFF technology, it has to be a heated template. The template is a very important part of every printer. This is the main reason researchers thought that the template could affect the printing accuracy.

More precisely, Sherman S.L. et al. looked over the difference in the accuracy of printed dental models when they were placed in different areas of the building template. A DLP printer was used in this study [24]. They placed the dental models in the middle of the template and on the corners. Their result suggested that the accuracy was not affected by the position of the printed model on the building template.

## 7. Design of the Base

There are different model base designs used in dentistry and, more specifically, in orthodontics. The most popular model base is the one based on the American Board of Orthodontics requirements. When the 3D printing technology became more involved in everyday practice, the horseshoe model base became very popular for the use of aligner fabrication. Another ability that is given with 3D printed technology is to print the model in a solid or hollow shape. All these factors could possibly affect the print by causing distortion in the dental model.

According to the literature, there are only two studies that examined the effect of these parameters on printed dental models. The first study was conducted by Camardella L.T. et al. in 2017 [28]. This article compared the ABO base design, the horseshoe design, and the horseshoe design with a posterior connection by using PPP and DLP technology. Their results suggested that the PPP printer was able to print accurately the model with all these different designs. DLP printer could not deliver an accurate result on the horseshoe design while it was really accurate for the ABO design and the horseshoe design supported with a posterior connection. The second study was performed by Sherman S.L. et al. in 2018 [24]. Their research focused on the comparison of a solid model base to a hollow model base by using DLP technology. They concluded that there are no statistically significant differences between the two different prints.

## 8. Printing Materials

The printing materials for the use of 3D printers are broadly classified based on their printing technologies. The most common technologies are the vat polymerization technologies (SLA, DLP, and LCD) use liquid photopolymers, including acrylates and epoxides. The 3D material extrusion technology (FFF) uses polylactic acid (PLA) or acrylonitrile butadiene styrene (ABS). PPP technology uses photopolymers resins (acrylates) in liquid form.

There is only one study that looked over the effect of printing material on the accuracy of printed dental models. This study was conducted by Pereira A.B. et al. in 2020 [22]. In this study, there were used two different DLP printers creating three different model groups for the evaluation of the material effect on the print. The two groups used the recommended resin according to the manufacturer’s suggestion, and the third one used a hand-held digital caliper resin. Their results suggested that the accuracy of the printed model was affected by the use of a different resin than the manufacturer’s choice. More accurately, there was a difference on the vertical plane when a thirds party resin was used.

## 9. Conclusions

The accuracy of three-dimensional printed models can be affected by the different 3D printing technologies. According to the present systematic review, the most accurate technology of all is the PPP, followed by the DLP, LCD, Laser-SLA, and FFF printing technology, respectively. Another factor that seems to affect the accuracy of 3D dental models is the design of the base. It is proven that the horseshoe design could be distorted while the regular dental model base and a horseshoe model with a posterior connection are accurate. The last factor that affects the 3D printed dental models’ accuracy is the printing materials used. The accuracy of the 3D printed dental models is not affected by the layer height or the position of the model on the building template. There is no difference in the accuracy of the dental model’s print, whether the shape of choice is solid or hollow. All these factors do not affect the clinical orthodontic outcome. Since there is no clinically significant effect on the clinical outcome from the above-mentioned factors, the choice of printer for the clinicians can be guided according to the cost and time consumption to produce the dental models for orthodontic purposes. Finally, there must be more studies in order to have a strong scientific-based conclusion about this type of technology.

## Figures and Tables

**Table 1:** The search strategy for PubMed.

“Orthodontics” [Majr] and dental model	3405 results
“Models, Dental” [Majr] AND “Orthodontics”[Majr]	213 results
“Printing, Three-Dimensional” [Majr] AND Models, Dental”[Majr]	50 results
“Printing, Three-Dimensional” [Majr] AND “Orthodontics”[Majr]	35 results

**Table 2:** Inclusion and exclusion criteria.

Inclusion Criteria	Exclusion Criteria
Studies that refer to 3D printing technology in orthodontics for dental models	Studies that are reviews or authors’ opinion
In vitro studies prospective or retrospective	

**Table 3:** Data extraction.

Authors/ Publication Year	Study Design	Participants (Number of Dental Models)	Intervention	Outcomes	Method of Outcome Assessment	Results	Conclusion
Hazeveld A [30] (2014)	In vitroprospective	12 mandibular and maxillary models	2 types of printers DLP PPP	Accuracy and reproducibility of 3D printed models	Linear measurements	Clinical crowns: PPP > DLP Width of the teeth: DLP > PPP	Both 3D printers result in clinically acceptable dental models
Camardella LT [28] (2017)	In vitroprospective	10 pairs of printed dental models	Printers: SLA, PPP Three types of model base: regular horseshoe-shaped horseshoe-shaped with a bar	Accuracy of printers with different model base	- Ortho-Analyzer - Best fit algorithm software (Geomagic Qualify software)	PPP accurate regardless of the model base design SLA accurate only for regular and horseshoe-shaped with a bar	Regular base and horseshoe-shaped with a bar accurate regardless of the type of printer
Dietrich CA et al. [29] (2017)	In vitroprospective	2 different maxillary dentition casts (20 reproduced casts)	Printers: SLA PPP	Accuracy of SLA and PPP for dental models printing	Best fit algorithm software (IFM software)	Trueness: PPP > SLA Precision: SLA > PPP	PPP has better trueness, and SLA has better precision
Kim SY et al. [21] (2018)	In vitroprospective	A pair of typodont printed 5 times	4 types of printers SLA DLP FFF PPP	Precision and trueness of dental models printing	Half ball markers and 3D inspection software	Precision: PPP > DLP > SLA > FFF Trueness: PPP > SLA > DLP > FFF	PPP and DLP are more precise than other printers, while PPP has the highest accuracy
Park ME et al. [27] (2018)	In vitroprospective	10 printed models (1 master model)	Printers: PPP DLP	Accuracy and reproducibility of printing models vs. conventional stone models	Scanned with model scanner and Superiposition specialized software.	Stone models showed overall smaller volumetric changes PPP showed smaller volumetric changes than DLP	Conventional method is more reliable
Brown GB et al. [26] (2018)	In vitroprospective	30 pair of dental casts	Printers: PPP DLP	Accuracy and reproducibility of printing models vs. conventional stone models	Scanned with model scanner and digital linear measurements	All measurements were high reproducible No differences on all linear measurements except the crown height between the stone model and DLP printer	Both DLP and PPP are clinically acceptable
Loflin WA et al. [25] (2019)	In vitroprospective	12 sets of final orthodontic models	3 different layer heights: - 25 μm - 50 μm - 100 μm	Effect of layer height on 3D printed models	Cast-Radiograph Evaluation grading system.	No statistically significant effects of print layer height 3D-printed models of each layer height were highly positively correlated with stone models	100 μm layer height 3D-printed models are potentially clinically acceptable
Sherman SL et al. [24] (2020)	In vitroprospective	15 pairs of dental casts for each measurement	DLP printer - placement on the build plate (middle vs. corner) - thickness in the *z*-axis (50 microns vs. 100 microns), - hollow vs. solid shell	Accuracy of DLP on position Accuracy of DLP on layer height Accuracy of DLP on base	Linear measurements on the dental arch	No difference in the plate position No difference in layer height No difference in the model base	DLP printer produced clinically acceptable models
Akyalcin S. et al. [23] (2021)	In vitroprospective	20 pairs of dental casts with ABO Index Between 10–30	3 types of printers SLA DLP PPP	Linear and surface accuracy of dental models fabricated using 3 different 3D printers	Linear measurements and Best fit algorithm software	PPP models produced significantly less surface variation than the DLS and SLA models	The differences between the printers are not likely to be clinically significant for orthodontic applications
Lo Giudice A et al. [31] (2022)	In vitroprospective	1 master digital dental model	2 LCD printers and 1 SLA printer	Dental and skeletal measurements	Surface-based superimposition	RMS values detected were significantly higher in dental models prototyped with entry-level compared to the SLA printer No significant differences were found between the values of RMS of both entry-level 3D printers Layer thickness did not affect either the trueness or precision of the 3D-printed models	Entry-level LCD-based 3D printers are not as accurate as Professional-grade 3D printer, but still close to orthodontics clinical threshold values
Pereira ABN et al. [22] (2022)	In vitroprospective	14 dental models	3 different DLP printers FFF PPP	Accuracy, precision, and time consumption of 3D printers with different cost	Model superimposition (Geomagic Qualify software)	The results showed that all printers produced similar results FFF has the cheapest model production The PPP printer was considered the fastest	DLP printers were considered the best cost–benefit ratio for small independent dental offices

**Table 4:** Risk of bias assessment.

Author (Year)	Outcomes	Bias Due to Confounding	Bias in Selection of Participants in the Study	Bias in Measurement of Interventions	Bias Due to Departures from Intended Interventions	Bias Due to Missing Data	Bias in Measurement of Outcomes	Bias in Selection of the Reported Result	Overall Bias
Hazeveld A [30] (2014)	Accuracy and reproducibility of 3D printed models	Low for all outcomes	Low for all outcomes	Low for all outcomes	Low for all outcomes	Low for all outcomes	Low for all outcomes	Low for all outcomes	Low for all outcomes
Camardella LT [28] (2017)	Accuracy of printers with different model base	Low for all outcomes	Low for all outcomes	Low for all outcomes	Low for all outcomes	Low for all outcomes	Low for all outcomes	Low for all outcomes	Low for all outcomes
Dietrich CA et al. [29] (2017)	Accuracy of SLA and PPP for dental models printing	Low for all outcomes	Low for all outcomes	Low for all outcomes	Low for all outcomes	Low for all outcomes	Low for all outcomes	Low for all outcomes	Low for all outcomes
Kim SY et al. [21] (2018)	Precision and trueness of dental models printing	Low for all outcomes	Low for all outcomes	Low for all outcomes	Low for all outcomes	Low for all outcomes	Low for all outcomes	Low for all outcomes	Low for all outcomes
Park ME et al. [27] (2018)	Accuracy and reproducibility of printing models vs. conventional stone models	Low for all outcomes	Low for all outcomes	Low for all outcomes	Low for all outcomes	Low for all outcomes	Low for all outcomes	Low for all outcomes	Low for all outcomes
Brown GB et al. [26] (2018)	Accuracy and reproducibility of printing models vs. conventional stone models	Low for all outcomes	Low for all outcomes	Low for all outcomes	Low for all outcomes	Low for all outcomes	Low for all outcomes	Low for all outcomes	Low for all outcomes
Loflin WA et al. [25] (2019)	Effect of layer height on 3D printed models	Low for all outcomes	Low for all outcomes	Low for all outcomes	Low for all outcomes	Low for all outcomes	Low for all outcomes	Low for all outcomes	Low for all outcomes
Sherman SL et al. [24] (2020)	Accuracy of DLP on position Accuracy of DLP on layer height Accuracy of DLP on base	Low for all outcomes	Low for all outcomes	Low for all outcomes	Low for all outcomes	Low for all outcomes	Low for all outcomes	Low for all outcomes	Low for all outcomes
Akyalcin S. et al. [23] (2021)	Linear and surface accuracy of dental models fabricated using 3 different 3D printers	Low for all outcomes	Low for all outcomes	Low for all outcomes	Low for all outcomes	Low for all outcomes	Low for all outcomes	Low for all outcomes	Low for all outcomes
Lo Giudice A et al. [31] (2022)	Dental and skeletal measurements	Low for all outcomes	Low for all outcomes	Low for all outcomes	Low for all outcomes	Low for all outcomes	Low for all outcomes	Low for all outcomes	Low for all outcomes
Pereira ABN et al. [22] (2022)	Accuracy, precision and time consumption of 3D printers with different cost	Low for all outcomes	Low for all outcomes	Low for all outcomes	Low for all outcomes	Low for all outcomes	Low for all outcomes	Low for all outcomes	Low for all outcomes

## Data Availability

Not applicable.
